# Revisiting Montreal: New Insights into Symptoms and Their Causes, and Implications for the Future of GERD

**DOI:** 10.1038/s41395-018-0287-1

**Published:** 2018-10-15

**Authors:** A. Pali S. Hungin, Michael Molloy-Bland, Carmelo Scarpignato

**Affiliations:** 1 The Institute of Health and Society, Faculty of Medical Sciences, Newcastle University, Newcastle upon Tyne, UK;; 2 Research Evaluation Unit, Oxford PharmaGenesis, Oxford, UK;; 3 Clinical Pharmacology & Digestive Pathophysiology Unit, Department of Clinical & Experimental Medicine, University of Parma, Parma, Italy.

## Abstract

The Montreal definition of gastroesophageal reflux disease (GERD) provided a rationale for acid suppression medication without investigation, thus enhancing the management of the substantial symptom burden in these patients. Increased proton-pump inhibitor use has also highlighted their limitations, with one third of “typical” symptoms known to be refractory. Most refractory symptoms are ascribed to reflux hypersensitivity (RH) and functional heartburn (FH). RH may be caused by impaired esophageal mucosal barrier function and sensitization of peripheral esophageal receptors. Central sensitization may also contribute to the perception of non-pathologic reflux in RH, and the perception of physiological stimuli in FH. Importantly, mechanisms underlying GERD, RH, and FH are (in theory) not mutually exclusive, further complicating patient management. Methods used to distinguish GERD from RH and FH are impractical for use in epidemiological studies and pragmatic care and may have limited diagnostic accuracy. This is impeding accurate prevalence estimates and risk factor determination and the identification of new therapies. Direct assessment of mucosal barrier function by measuring impedance is a promising candidate for improved diagnosis. Ultimately though the concept of GERD as a composite, symptom-based entity needs re-evaluation, so that new understandings of upper GI symptoms can direct more precise management.

## INTRODUCTION

The primary goal of this review was to revisit the concept of gastroesophageal reflux disease (GERD). The previously variable definition of GERD was finally standardized in 2006 with the Montreal Consensus, which states that GERD is “a condition that develops when the reflux of stomach contents into the esophagus causes troublesome symptoms and/or complications” (1). The Montreal Definition of GERD encapsulates most eventualities, from patients with reflux esophagitis with or without symptoms (the latter actually accounting for close to 40% (2) of such patients in Western populations, and possibly a much higher proportion in Asian populations (3)) to those with symptoms but no other findings. The latter, purely symptom-based definition of GERD provided a rationale for treatment with acid-suppressive medications without the need for cumbersome investigations of symptom etiology, thus enhancing management of the substantial symptom burden in these patients (4–6). The Montreal Definition also acknowledges that reflux contents other than acid can cause symptoms. However, the degree of the complexity of the relationship between symptoms and reflux was perhaps not fully appreciated at the time of its development. The situation has been further complicated by the subsequent, comprehensive categorization of a range of functional esophageal disorders that are indistinguishable from GERD without substantial investigation (7). As part of our revisiting the concept of GERD, we recap our current understanding of the relationship between suspected GERD symptoms and reflux (or lack thereof), with a focus on the persisting clinical challenge and possible mechanisms of reflux hypersensitivity and functional heartburn. Finally, we discuss the implications of these findings for our current assumptions about suspected GERD symptoms, including whether treating GERD as a composite disease entity may now be a barrier to more precise patient management.

## ARE TYPICAL SYMPTOMS INDICATIVE OF ACID REFLUX?

Even the “typical” symptoms of GERD, defined in the Montreal Consensus as heartburn and regurgitation, may not be that typical, with their diagnostic value questioned in the Diamond study (8). In this study, consecutive patients presenting to their family practitioner with upper gastrointestinal (GI) symptoms were asked to identify their most troublesome symptom. These data were then analyzed according to whether or not patients had GERD based on objective investigation, defined as either the presence of reflux esophagitis, pathologic esophageal acid exposure (pH > 4 for > 5.5% of the time over a 24 h period), or a positive symptom association probability (SAP) for acid reflux. Tellingly, only 49% of patients with objectively defined GERD selected heartburn or regurgitation as their most troublesome symptom. Furthermore, the use of esomeprazole was found to be neither sensitive nor specific for the diagnosis of GERD in this study, clearly challenging the previously held adage that a proton pump inhibitor (PPI) response test could help to distinguish patients with GERD from those with other conditions. This is disappointing, though not necessarily surprising. Evidence from a recent study of 2665 individuals from Russia, Brazil, the UK, and USA, in which patients were asked to describe symptoms in their own words, suggest that heartburn (as interpreted by clinicians) may encompass at least two distinct symptoms, potentially with different etiologies (9). Indeed, the complex etiology of presumed reflux-induced symptoms is at the heart of issues with PPI response.

## REFLUX–SYMPTOM RELATIONSHIPS AND PPI RESPONSE

The first indication that not all suspected GERD symptoms are caused by acid reflux came from Sifrim et al. in 1999, with the application of 24-h esophageal pH and multichannel intraluminal impedance monitoring in an experimental setting. This allowed reflux episodes to be detected regardless of their pH, and it was noted that only about half of reflux episodes detected in normal subjects using impedance monitoring were also detected by pH-metry (10).

The majority of studies that actually quantify the extent to which non-acid (weak acid or weak alkaline) reflux contributes to symptoms in patients with GERD came after the Montreal Consensus. These were recently reviewed to show that nearly a third (28%) of reflux-related symptoms are associated with non-acid reflux (11). Similarly, a study by Savarino et al. found that in 87 (38%) of 226 patients with heartburn and/or regurgitation occurring at least three times a week, a positive association between reflux and symptoms (SAP > 95% (12)) was only observed if nonacid reflux was also accounted for (13). A positive SAP could not be achieved for any type of reflux in around a fifth of patients in this study (13).

**Fig. 1:**
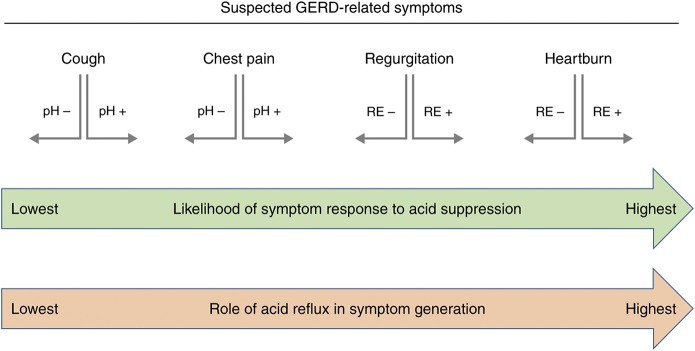
Suspected gastroesophageal reflux disease (GERD)-related symptoms, their relative likelihood of response to acid-suppressive therapy based on the literature, and the presumed importance of acid reflux for symptom generation based on these findings. Smaller arrows underneath each symptom indicate the direction of the shift in response to acid suppression when patients are selected based on the presence (RE+) or absence (RE−) of reflux esophagitis, or the presence (pH+) or absence (pH−) of pathologic esophageal acid exposure. The relative position of each symptom and the size of the arrows is not to scale. In summary, heartburn is assumed to be the quintessential acid-related symptom and as such correlates the most frequently with pathologic esophageal acid exposure and response to acid-suppressive therapy. The response of heartburn (and regurgitation) to acid suppression is higher in RE+ patients than in RE− patients, concordant with RE being a good proxy for pathologic esophageal acid exposure in lieu of pH-testing. The relative response of symptoms to acid suppression decreases as their dependence on pathologic esophageal acid exposure decreases. However, even for symptoms such as chest pain and cough, response rates to acid suppression can be enhanced by identifying patients who have pathologic esophageal acid exposure (pH+), albeit with a dwindling effect as symptom etiology becomes increasingly multifactorial

The relative response of different symptoms to acid suppression therapy correlates with their dependence on acid (Fig. 1). While heartburn is more responsive than regurgitation to PPIs (14), both respond better to PPIs than chest pain, for which the therapeutic response is minimal in unselected individuals (50% relief in 0–17% of patients) (15). However, chest pain responsiveness to PPIs increases substantially (50% relief in 56–85% of patients) if those with pathologic esophageal acid exposure are selected, indicating that acid reflux can be a major contributor to this symptom, but that it is one of many possible causes (15). Selecting patients with pathologic esophageal acid exposure also appears to improve the therapeutic gain of PPIs for chronic cough, though the shift in efficacy is very modest (from 0–9% to 12–36%) indicating that mechanisms behind this very common and fairly generic symptom are even more heterogeneous than for chest pain, and largely unrelated to acid (16).

Given the above, it is not surprising that the proportion of reflux-related symptoms associated with non-acid reflux jumps drastically to around 80% in patients who experience symptoms despite taking a PPI (11). A similarly dramatic increase in symptoms not related to any type of reflux is also observed, as shown in the seminal paper by Mainie et al. in which less than half of symptoms in 200 patients taking a PPI twice daily were related to reflux (8% acid and 35% non-acid) (17). It should be noted that these figures incorporate both typical and atypical symptoms of GERD. Nevertheless, the proportion of patients who had a positive SAP for acid or non-acid reflux was low for the typical GERD symptoms of unexplained chest pain (18%), heartburn (30%), and regurgitation (52%) (17).

**Fig. 2:**
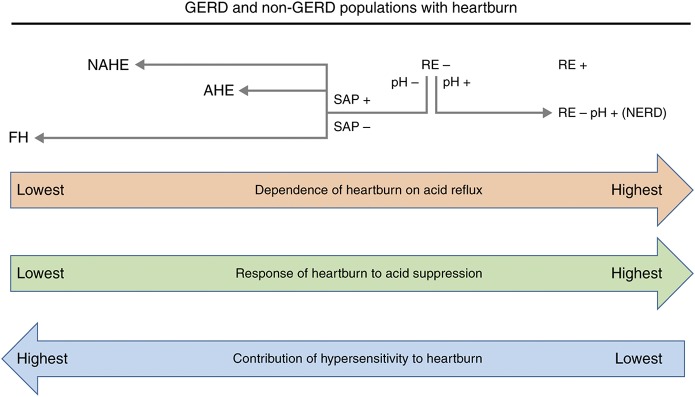
Relationship between reflux acidity, response of heartburn to acid-suppressive therapy and the role of peripheral and/or central esophageal hypersensitivity in different gastroesophageal reflux disease (GERD) and non-GERD patients with heartburn. Reflux esophagitis (RE) correlates strongly with the presence of pathologic esophageal acid exposure and, concomitantly, healing of RE and heartburn resolution are high with acid-suppressive therapy in these patients. Some patients have heartburn but not RE. Those who have pathologic esophageal acid exposure (pH+) have NERD, and heartburn symptoms respond as well to acid suppression as they do in patients with RE. Those patients with heartburn who do not have pathologic esophageal acid exposure may still have a positive symptom association probability (SAP+) for acid or non-acid reflux, and are thus categorized as having reflux hypersensitivity (acid hypersensitive esophagus (AHE) or non-acid hypersensitive esophagus (NAHE)). Patients without pathologic acid exposure who have a negative SAP are designated as having functional heartburn (FH). The role of peripheral and/or central esophageal hypersensitivity increases as dependence on acid reflux (and response to acid suppression) decreases, in line with heartburn perception occurring despite non-pathologic acid reflux (hyperalgesia) in patients with reflux hypersensitivity, or under physiological reflux conditions (allodynia) in patients with FH

The relationship between sub-types of GERD and non-GERD conditions, reflux acidity, esophageal hypersensitivity (discussed later), and response to PPIs is shown in Fig. 2. Reflux is more often acidic in patients with reflux esophagitis than in those without (13,18,19), consistent with the strong causal link between reflux esophagitis and esophageal acid exposure (20). Reflux esophagitis is also, as one might expect, the most responsive of all GERD manifestations to acid suppression with PPIs (21,22), with healing rates of over 80% (and possibly higher with prolonged treatment (23)) (24). Selecting patients with reflux esophagitis strongly selects for those with pathologic esophageal acid exposure. As such, the symptoms experienced by patients with reflux esophagitis are more often related to acid and thus more responsive to PPIs than symptoms in patients without reflux esophagitis (13,25,26). In addition, lower esophageal sensitivity to acid in patients with reflux esophagitis (particularly high grade) than in those with NERD (27) may lower the degree of acid suppression required to alleviate heartburn, also contributing to better PPI response. However, when patients without reflux esophagitis who have pathologic esophageal acid exposure are selected, the estimated complete symptom response rate with PPIs is comparable to that for reflux esophagitis (28).

Agents that have been shown to have some benefit in patients with PPI-resistant symptoms include alginates, mucosal protective agents, and baclofen. Alginates precipitate into a low-density viscous gel and have been shown to reduce the burden of reflux symptoms in patients with residual symptoms despite PPI use (29). This may be because they can displace the acid pocket—an unbuffered zone of acid that forms postprandially in the stomach, and which may not completely disappear with PPIs (30,31). Addition of a mucosal protection agent to PPI treatment has been shown to significantly improve symptoms and health-related quality of life compared with PPI alone in patients with NERD (32). Furthermore, baclofen was found to reduce the number of reflux episodes (but not acid reflux episodes) in patients with PPI-resistant symptoms *via* inhibition of transient lower esophageal sphincter relaxations (33), and to improve persistent symptoms associated with non-acid duodenal reflux in patients that were refractory to PPI treatment (34). The therapeutic benefits of these drugs in patients with PPI-resistant symptoms may relate to their effects on residual pathologic acid, or on symptoms caused by non-pathologic acid or non-acid reflux due to reflux hypersensitivity. Indeed, reflux hypersensitivity and functional heartburn are thought to account for most pain-related symptoms that persist despite PPI therapy.

## DIAGNOSIS OF REFLUX HYPERSENSITIVITY AND FUNCTIONAL HEARTBURN

As highlighted in a landmark review by Savarino et al., the common practice of identifying “NERD” patients solely on the basis of typical symptoms and an absence of reflux esophagitis selects for a heterogeneous patient group (35), of which only those where acid is implicated in symptom generation are clearly responsive to PPIs (36). These groups consist of those with esophageal acid exposure (true NERD), normal esophageal acid exposure but a positive SAP for acid reflux (acid hypersensitive esophagus) or non-acid reflux (non-acid hypersensitive esophagus) and those with normal esophageal acid exposure and a negative SAP for any type of reflux (functional heartburn) (35). This new understanding is reflected in the most recent iteration of the Rome criteria (Rome IV) for functional esophageal disorders, in which the acid hypersensitive esophagus and non-acid hypersensitive esophagus groups have been combined into the new category of reflux hypersensitivity, that would appear to fit somewhere between NERD and functional heartburn (7).

It should be noted that the SAP method was developed and validated in PPI-naive patients (12). In the first study to determine the utility of the SAP method in patients with PPI-refractory symptoms (in whom it is most commonly employed in the clinic) it was determined that at reflux rates of <10%, SAP values were largely determined by chance, rather than by the relationship between symptoms and reflux. The same study also found that around 70% of patients who are refractory to PPIs have a reflux rate of <10% (37). A more recent study by Choski et al. included a sub-group of patients with heartburn or regurgitation as their chief, PPI-refractory symptom (38). In this study, the difference between patients diagnosed with acid reflux hypersensitivity and those diagnosed with functional heartburn, in terms of the number of symptoms associated with reflux, equated to only 1‒2 pressings per day of the symptom reporting button (38). The authors did not include nonacid reflux in their assessments, pointing out that this increases the probability of chance associations between symptoms and reflux episodes (38). Other factors that could influence the accuracy of SAP include the shorter duration of impedance-detected reflux events versus acid-detected events (39), longer lag times for symptom perception after weakly acidic versus acid reflux (40), the potential for unreliable reporting of symptom timing by patients (41) and day-to-day variation in the onset of symptoms (38).

It remains to be seen whether reflux hypersensitivity should really be included as a functional esophageal disorder, given that its definition requires symptoms to be associated with reflux events (7). This will likely be an area for future debate. For now though, only the mechanisms that may contribute to reflux hypersensitivity and functional heartburn will be discussed.

## MECHANISMS OF REFLUX HYPERSENSITIVITY AND FUNCTIONAL HEARTBURN

Individuals with reflux hypersensitivity may have impaired esophageal functioning compared with those with functional heartburn. In one study, the prevalence of microscopic esophagitis, a potential marker of reduced mucosal integrity, was 65% in patients with reflux hypersensitivity, compared to 13% in patients with functional heartburn, and 15% in healthy volunteers (42). In another study, esophageal mucosal integrity and the rate of esophageal chemical clearance were both reduced in PPI-responsive patients with reflux hypersensitivity compared with patients with functional heartburn and healthy volunteers (43).

The prevalence of microscopic esophagitis in patients with functional heartburn is similar to the rate in healthy volunteers (42). The rate of proximal reflux events and weak-acid reflux events is also similar in patients with functional heartburn and healthy volunteers, but higher in those with reflux hypersensitivity (44). These data reinforce the lack of any detectable, organic origins of functional heartburn symptoms. Interestingly, microscopic esophagitis is more prevalent, and esophageal mucosal integrity and chemical clearance more greatly impaired in patients with GERD than in those with reflux hypersensitivity (42, 43). These findings indicate that patients with reflux hypersensitivity experience heartburn despite comparatively (vs NERD and reflux esophagitis) greater mucosal protection of their esophageal receptors from chemical stimuli, invoking mechanisms of enhanced receptor sensitivity.

### Peripheral and central hypersensitivity

Greater esophageal sensitivity to acid perfusion has been observed in patients with NERD compared with those with reflux esophagitis (27,45). Patients with GERD and those with functional heartburn have also been shown to be more sensitive to esophageal balloon distension than healthy controls (46,47). Interestingly, several receptors theorized to be involved in transducing pain signals in response to acid are also sensitive to mechanical distention (48). Esophageal distension caused by high reflux volume may therefore enhance the sensitivity of peripheral esophageal receptors to acid. Consistent with this, esophageal sensitivity to distension is enhanced in patients with GERD after perfusion of the esophagus with acid (47) and reflux symptom perception is enhanced in patients with NERD when gas (which enhances reflux volume) is present in reflux (49). Repeated activation of peripheral esophageal receptors may also cause central sensitization of neurons in the dorsal horn of the spinal cord (central hypersensitivity), leading to both hyperalgesia (increased sensitivity to pain cause by noxious stimuli) and allodynia (pain generated in response to physiological stimuli that do not normally cause pain) (50). Central sensitization can lead to changes in the sensitivity of areas remote from the site of the initial acid exposure (secondary hypersensitivity) (50–52). For example, acid perfusion into the lower esophagus has been shown to reduce the pain threshold for subsequent electrical stimulation of the chest wall to a greater extent in patients with non-cardiac chest pain than in those without (52). Secondary hypersensitivity may also contribute to the high prevalence of dyspeptic symptoms (around 40% (53)) in patients with GERD, as it provides a mechanism whereby gastroesophageal reflux may contribute to epigastric pain despite the latter being perceived as remote from the site of esophageal stimulation. Interestingly, dietary components may also play a role in central sensitization, with enhanced heartburn perception observed in one study after perfusion of fat into the duodenum, consistent with frequent reports by patients that diets high in fat worsen their symptoms (54).

Because central hypersensitivity (more than peripheral hypersensitivity) is thought to contribute to allodynia (55), it may be of relevance to functional heartburn, which is by definition due to inappropriate pain responses to stimuli that are within the normal, physiological range. Evidence that central sensitization, whether caused by previous acid-related injury or other predisposing factors, may contribute to functional heartburn, comes from its overlap with functional disorders in other parts of the gut, such as irritable bowel syndrome and functional dyspeptic symptoms (56–58). In a recent post hoc analysis of pooled clinical trial data, the response of “substernal burning” to PPIs was found to be reduced if “dyspeptic-pain” was also present, possibly because the overlap of symptoms from different locations hints at a shared central sensitization (rather than acid reflux)-dominant etiology (59). In addition, there is substantial laboratory-based evidence that symptoms experienced by patients with functional GI disorders can be induced or exacerbated with the administration of lipids (60).

A single dose of the selective serotonin reuptake inhibitor (SSRI) citalopram has been shown to reduce esophageal sensitivity to balloon distention and acid perfusion in healthy volunteers with established esophageal sensitivity (61). SSRIs have been shown to significantly reduce symptoms compared with placebo in patients with reflux hypersensitivity (citalopram (62)) and patients with reflux hypersensitivity or functional heartburn (fluoxetine (63)) who are refractory to PPI treatment. In the only study to assess a tricyclic anti-depressant (imipramine) in patients with reflux hypersensitivity and functional heartburn, quality of life significantly improved compared with placebo, but symptoms did not (64). Histamine-2 receptor antagonists (H_2_RAs) have also been shown to modulate esophageal sensitivity to acid in patients with GERD (65), and patients with functional heartburn (66).

### Psychological factors

The influence of the central nervous system extends to higher brain functions, with studies showing that psychological factors can increase the perception of heartburn (auditory stress (67) and life stress (68)) and esophageal pain (sleep deprivation (69)). Psychiatric comorbidity is high among patients with functional dyspepsia (70) and functional heartburn (71). Indeed, it cannot be ruled out that SSRIs may exert some of their effects on heartburn perception (discussed above) indirectly, *via* their effects on anxiety, mood, and sleep.

### Mucosal barrier function

Microscopic esophagitis, as the name implies, refers to the presence of esophageal lesions that are only visible upon microscopic (rather than macroscopic, i.e., endoscopy) investigation. The presence of microscopic esophagitis may be a marker of impaired mucosal integrity due to chronic exposure of the esophageal mucosa to noxious reflux components, and thus also a marker of symptoms that are related to reflux. Studies have reported the ability to distinguish between patients with GERD (with heartburn) and those without (72), and between patients with NERD and those with functional heartburn (42), using global histology scoring systems that combine the evaluation of a number of microscopic esophageal lesions (necrosis/erosion, neutrophil/ eosinophil intraepithelial infiltration, basal cell hyperplasia, elongation of papillae, dilated intercellular spaces (DIS)). The main implication of these findings is that microscopic lesions may be a marker for impaired mucosal barrier function, which increases the exposure of peripheral esophageal receptors to reflux. In support of this pathophysiological mechanism, addition of a mucosal protection agent (a hyaluronic acid-chondroitin sulphate-based bioadhesive formulation) to PPI treatment was recently shown to improve symptoms and health-related quality of life to a significantly greater extent than PPI alone in patients with NERD (32). The idea that variation in the degree of exposure of peripheral receptors to refluxate contributes to symptom perception is further supported by the recent observation that nociceptive sensory nerves are closer to the surface in the proximal and distal esophagus of patients with NERD than in controls or patients with reflux esophagitis or Barrett’s esophagus (73). An earlier study by the same group found these nerve fibers were closer to the surface at the proximal esophagus than at the distal esophagus (74), consistent with the association of more proximal (vs distal) reflux with typical GERD symptoms (75–77).

Impaired esophageal mucosal barrier function has been shown to occur with DIS formation in response to acid or bile acids (78,79). DIS formation and enhanced permeability of the esophageal mucosal barrier occurs more readily in patients with NERD than in controls, suggesting an inherent susceptibility (79). Furthermore, while the resolution of DIS generally occurs in patients whose symptoms respond to PPI therapy, they tend to persist more in non-responders (80). Increased penetration of weakly acidic or even alkaline reflux into esophageal tissue may mean that more receptors are exposed for longer, increasing the likelihood that symptom perception thresholds are reached. This is consistent with longer lag times for symptom perception following weakly acidic reflux compared with acid reflux (40). It is interesting that stress, in addition to possibly increasing the perception of symptoms, may also induce DIS formation in the esophageal mucosa (81).

In the most recent (and largest) study to assess the diagnostic value of esophageal histology, total epithelial thickness was the best-performing criterion for identifying patients with investigation-defined GERD, and was also able to identify patients with NERD, reflux esophagitis, and pathologic esophageal acid exposure (82). DIS was not found to be a significant predictor of GERD. However, it is important to note that one of the main objectives of this study was to assess the level of agreement between assessors at the two pathology centres where the histologic examinations were performed using a refined global scoring system. In addition to being the best diagnostic criterion, total epithelial thickness also had the best inter-observer agreement, suggesting that the success of this lesion in identifying patients with GERD may be at least partially due to the degree of reliability with which it can be assessed, rather than necessarily being more indicative of impaired mucosal status than other lesions. Total epithelial thickness has subsequently been shown by the same group to enhance the diagnosis of GERD, particularly when concomitant epigastric pain is absent (83).

The inherent difficulties of identifying histologic markers that are both indicative of an impaired esophageal mucosal barrier and which can be assessed consistently across clinics, could be mooted by techniques that allow direct assessment of mucosal integrity. Impedance technology typically used to characterize reflux has been adapted to determine the baseline impedance of the esophageal mucosa, a measure that correlates with transepithelial resistance (84). Both prolonged acid exposure (85,86) and DIS (85,87) have been associated with lower esophageal baseline impedance. Furthermore, it has been shown that baseline impedance is lower in patients with GERD (NERD and reflux esophagitis) than in healthy volunteers (84) and can be used to distinguish patients with reflux esophagitis or NERD from those with functional heartburn (87). Using baseline impedance overcomes several limitations associated with the SAP, because accuracy should not be affected by variation in the reflux rate or temporal relationship between symptoms and reflux events, or the reliability of symptom reporting. Unfortunately, this approach is costly, cumbersome, and uncomfortable for the patient. However, more practical methods are being developed, at least two of which have shown high specificity and sensitivity for the diagnosis of GERD (88,89). It has also been suggested that baseline impedance could be useful for identifying patients in whom extra-esophageal symptoms are reflux related (90). Indeed, there is a desperate need for diagnostic methods that can accurately identify patients with extra-esophageal GERD symptoms.

## CONCLUSIONS AND IMPLICATIONS

Our understanding of the processes that mediate symptom perception and our ability to diagnose and treat symptoms decline rapidly as we move away from the more familiar territory of pathologic acid exposure, and into the hazier world of reflux hypersensitivity (to non-pathologic acid and non-acid reflux) and functional heartburn. Although reflux hypersensitivity and functional heartburn are not technically GERD (7), the complex array of tests required to identify these conditions hinders their exclusion in pragmatic clinical practice (91), where they account for the majority of the substantial symptom burden that exists despite PPI use (35,92). Indeed, clinical trials of reflux inhibitor molecules developed to target symptoms that are refractory to PPIs have so far been thwarted, partly because symptom-based approaches to patient inclusion were used that do nothing to exclude patients with functional heartburn (93,94).

Despite the poor relationship of even the typical symptoms of heartburn and regurgitation to pathologic acid reflux, treatment of suspected GERD symptoms with a PPI remains the best initial management approach. However, when PPI-resistant symptoms are encountered, an appreciation of the heterogeneous causes of suspected GERD symptoms becomes paramount. Without this, consequences may include assumptions of low adherence to PPIs that create barriers to further investigation, and referrals for surgical interventions that may do little to address mechanisms that underlie functional heartburn, which at the moment can only be distinguished from NERD and reflux hypersensitivity with impedance–pH monitoring (35). It is easy to imagine that a symptom-based rationale for managing GERD, developed by expert consensus and reinforced by excellent (though probably overestimated by physicians (95)) response rates for PPIs, might have reduced the treating physician’s appreciation of other symptom etiologies, especially when (unlike for acid reflux) there are no targeted treatments to reinforce the validity of other mechanisms. It would be interesting to assess what impact (if any) the symptom-based Montreal Definition of GERD has had on physicians’ perceptions and management of symptoms that persist despite the use of PPIs.

Reflux hypersensitivity can theoretically involve mechanisms of impaired esophageal mucosal barrier function (leading to greater exposure of peripheral receptors to reflux components), sensitization of peripheral esophageal receptors, and central sensitization which may occur due to repeated activation and input from peripheral receptors, or because of top–down inputs (e.g., stress), in either case leading the enhanced perception of stimuli. Heartburn becomes functional (rather than due to reflux hypersensitivity) when it occurs in the presence of physiological levels of reflux, thus shifting the etiology towards enhanced perception rather than an enhanced stimulus. Based on this model, a patient with GERD could theoretically develop functional heartburn due to past, repeated activation of peripheral receptors by acid reflux. Upon treatment with a PPI, acid reflux may be resolved, but functional heartburn due to central sensitization may be unmasked. Indeed, there is no reason why any number of combinations of factors that contribute to symptoms could not exist in a single patient, and this consideration should be factored into management strategies.

Measuring symptoms is far easier to achieve on a large scale than the cumbersome physiological tests required to confirm if GERD is really present. As such, our most widely adopted assessments of the potential future prevalence of GERD are based on symptoms only (96). But what version of symptom-based GERD will we actually be treating in the future? There is substantial evidence that increases in the prevalence of GERD symptoms are largely associated with increasing rates of obesity (97). The most popular mechanistic view underlying this association is that abdominal obesity increases intra-abdominal pressure, which directly increases the propensity to reflux as well as promoting the development of hiatus hernia (98). However, there is also evidence that symptoms experienced by patients with functional GI diseases are enhanced by dietary lipids, and an increasing role for stress in functional heartburn (i.e., central sensitization) and reflux hypersensitivity (DIS and peripheral and central sensitization) also needs to be considered. Changes over time in the distribution of factors that contribute to the symptoms used to diagnose GERD would also be expected to change the relative efficacy of different therapeutic approaches.

In conclusion, our base concepts, as espoused within the Montreal Consensus, may now be a handicap in explaining and effectively managing patients. We need a greater understanding of symptoms based on patients’ descriptors rather than clinicians’ categories to generate new targets for drug development and/or guide potential new applications for existing (or even discarded) drugs. Pragmatic tools must be developed to distinguish patients with GERD from those with reflux hypersensitivity and functional heartburn, for use in clinical trials of new drugs, and so that population-based prevalence estimates and risk factors can be more accurately determined. Importantly, these tools must be sufficiently refined for use in pragmatic clinical practice, so it is feasible to target the appropriate drugs to the right patients. New methods for assessing esophageal mucosal integrity look promising, though other biomarkers (recently reviewed (99)) should continue to be investigated. However, even with such developments, the complexity of symptoms and causes requires that the concept of GERD as a composite, symptom-based entity be re-evaluated, if more precise patient management is to be achieved.

## CONFLICTS OF INTEREST

**Guarantor of the article:** A. Pali S. Hungin.

**Specific author contributions:** APSH developed the concept/ methodology and contributed to the content and drafting of the manuscript. CS and MM-B contributed to the content and drafting of the manuscript and MM-B also provided editorial oversight. All authors approved the final version for submission.

**Financial support:** This research was funded by an unrestricted grant from Reckitt Benckiser.

**Potential competing interests:** APSH has served on advisory Boards for Reckitt Benckiser, Allergan, and Danone and is a committee member of the Rome Foundation for Functional Gastrointestinal disorders. MM-B is a contract employee of Oxford PharmaGenesis^TM^ Ltd, Oxford, UK, which received a departmental grant for this project from the School of Medicine, Pharmacy and Health, Durham University, Durham, UK. CS is a member of the Speakers’ Bureau and of the Scientific Advisory Board of Reckitt-Benkiser, Alfa Wassermann and Sanofi.
